# Food-Pics_Extended—An Image Database for Experimental Research on Eating and Appetite: Additional Images, Normative Ratings and an Updated Review

**DOI:** 10.3389/fpsyg.2019.00307

**Published:** 2019-03-07

**Authors:** Jens Blechert, Anja Lender, Sarah Polk, Niko A. Busch, Kathrin Ohla

**Affiliations:** ^1^Department of Psychology, University of Salzburg, Salzburg, Austria; ^2^Centre for Cognitive Neuroscience, University of Salzburg, Salzburg, Austria; ^3^Department of Psychology and Education, Free University of Berlin, Berlin, Germany; ^4^Institute of Psychology, University of Münster, Münster, Germany; ^5^Research Center Jülich, Institute of Neuroscience and Medicine (INM-3), Cognitive Neuroscience, Jülich, Germany

**Keywords:** experimental research, image database, eating behavior, food stimuli, cue reactivity

## Abstract

Our current environment is characterized by the omnipresence of food cues. The taste and smell of real foods—but also graphical depictions of appetizing foods—can guide our eating behavior, for example, by eliciting food craving and anticipatory cephalic phase responses. To facilitate research into this so-called cue reactivity, several groups have compiled standardized food image sets. Yet, selecting the best subset of images for a specific research question can be difficult as images and image sets vary along several dimensions. In the present report, we review the strengths and weaknesses of popular food image sets to guide researchers during stimulus selection. Furthermore, we present a recent extension of our previously published database *food-pics*, which comprises an additional 328 food images from different countries to increase cross-cultural applicability. This *food-pics_extended* stimulus database, thus, encompasses and replaces *food-pics*. Normative data from a predominantly German-speaking sample are again presented as well as updated calculations of image characteristics.

## Introduction

Our current environment is characterized by frequent cues of highly palatable foods. Many researchers partially attribute rising obesity rates and problems in eating-related self-regulation to this factor (e.g., Davis et al., 2011). Today’s foods—processed as well as unprocessed—have reached a level of refinement that appeals strongly to our senses: visual, gustatory, olfactory, and oro-sensory food properties interact in creating hedonic pleasure. Pervasive advertisement penetrates real and virtual lives and constantly taxes self-regulation.

Research uses food images as experimental stimuli in a range of different paradigms. The food-viewing paradigm attempts to simulate environmental conditions in a controlled laboratory environment. Passive picture viewing is seen as a preparatory or anticipatory stage in food intake: natural eating settings often start with exposure to a food’s visual appearance along with its smell. Such preparatory stages are of interest to research as anticipatory cephalic phase responses might underlie conditioned food cravings (Berthoud and Morrison, 2008; Dagher, 2012). Passive picture viewing is not the only way in which food images are used: Pavlovian or operant conditioning setups pair foods with neutral images and thereby tap into learning (e.g., Blechert et al., 2016;Wardle et al., 2018), memory setups tap into retention (Meule et al., 2012), and lateral or non-foveal presentations investigate spatial attention (Castellanos et al., 2009). Research has repeatedly demonstrated that food images capture attention (Nummenmaa et al., 2011; Cunningham and Egeth, 2018), are prioritized during neural processing (Toepel et al., 2009; Meule et al., 2013), and consistently activate brain areas associated with reward, salience, and cognitive control (Dagher, 2012; Tang et al., 2012; Spence et al., 2016). These reward-related neural responses can be enhanced by both the presentation of energy-dense food (Killgore et al., 2003; Schur et al., 2009) and by manipulations of hunger (Uher et al., 2006; Fuhrer et al., 2008; Siep et al., 2009) or cravings (Pelchat et al., 2004). Furthermore, individuals with obesity (compared to healthy weight controls) show increased activation in reward-related brain regions induced by particularly energy-dense cues (Pursey et al., 2014). In their meta-analysis, Boswell and Kober (2016) showed that food cue reactivity and craving predicted eating and weight gain, and that the effect sizes of this prediction were similar for visual food cues and real food exposure (and stronger than those predicted by olfactory cues). This is an impressive demonstration of the power of visual food cues on appetitive responding and health. More recently, research has used food images to change associated evaluations and response tendencies, such as in motor response inhibition trainings (Stice et al., 2016; Jones et al., 2018).

The predominance of picture viewing in experimental research has brought about the need for adequate stimulus material. While earlier research had used food images from cookbooks, unspecified internet searches, or other databases such as the International Affective Picture System (IAPS), it soon became clear that these images yielded both insufficient images quality (e.g., poor resolution or contrast) and limited variance (e.g., in food categories, portion sizes, or viewing angles). As a result, considerable effort has been invested in the development of standardized, high quality, and open source materials. Several sets of pictures have been published recently, providing researchers with more options. Based on the IAPS, Miccoli et al. (2014) published the open library of affective foods (OLAF) with a particular focus of naturalistic settings. The macronutrient picture system (MAPS) is a relatively small set but provides detailed macronutrient composition for each food image (King et al., 2018). Larger image sets were presented by Foroni et al. (2013) [FoodCast Research Image Database (FRIDa)] and Charbonnier et al., 2016 [Food4Health (F4H); 2016], along with ratings from larger samples on various subjective properties such as energy density. Finally, *food-pics* (Blechert et al., 2014) was introduced by our group and includes a large number of images along with normative ratings and computational measures of image characteristics. However, requests from *food-pics* users to include further items motivated the search for additional images. For example, several food items popular in France, the United Kingdom, Austria, Germany, the Middle East, and Asia, a wider range of baked goods (e.g., different kinds of dark bread), a wider range of portion sizes for fruits and vegetables (including single foods and sliced fruits), as well as drinks were added. Also, improvements to the indices of image characteristics were made.

Existing image sets vary on several dimensions that might be of relevance to researchers looking for experimental stimuli. Hence, in addition to describing the extension of the *food-pics* database (aim *i*), the present report reviews other food image datasets popular in experimental research (aim *ii*), and assesses their strengths and weaknesses in order to guide researchers during the selection of the optimal database (aim *iii*). Toward this end, we pay particular attention to properties such as database size and intercultural applicability, the existence of normative data and computational measures of image characteristics, and the range and coverage of various food types and settings (single foods, solid foods vs. drinks, main meals vs. snacks, naturalistic vs. highly controlled settings). We review image sets that were freely available and established for the purpose of experimental picture-viewing paradigms in humans. Image sets established for the development and training of automatic recognition algorithms [e.g., Pittsburgh Fast-Food Image Dataset, Chen et al., 2009; University of Catania (UNICT) Food Dataset 889, Farinella et al., 2015; the ChineseFoodNet, Chen et al., 2017] are not reviewed, as they serve a different purpose. More generally, the present report aims to facilitate comparability and replicability of food-related research on the level of experimental stimuli.

## Materials and Methods

### Food-Pics_Extended

#### Stimuli

The extended *food-pics* database added 328 food images to the original 568 images (for details see Blechert et al., 2014). Images were provided by several researchers using *food-pics*^1^. Categories of foods include sweet (e.g., banana split), savory (e.g., ravioli), processed (e.g., fried chicken), and whole (e.g., orange) foods as well as beverages (e.g., milk). Several single images of individually presented foods were added to allow for a relatively precise estimation of nutritional composition and calorie content compared to foods that consist of several components. As in the original dataset, images comprised both single items (e.g., 1 blackberry) and numerous items (e.g., 11 blackberries) as well as meals (e.g., salmon and spinach). The same non-food items as previously described by Blechert et al. (2014) were included in the extended database for obtaining comparable normative ratings. For standardization, all images were edited onto a white background and homogenized according to viewing distance (≈80 cm), angle, and simple figure-ground composition. Plates and bowls were shown when necessary (e.g., ice cream sundae), though most foods could be presented without (e.g., fruits).

#### Image Characteristics

Image properties characterizing the images’ physical properties were computed using customized MATLAB scripts (The Mathworks, Inc. Natick, United States), which can be downloaded from the *food-pics* website^2^. A full description of image characteristic analysis is provided in the original report (Blechert et al., 2014). In brief, *size* was quantified as the proportion of non-white pixels. *Color* properties were quantified as the contribution of red, green, and blue color channels to the non-white pixels. Within-object *contrast* was quantified as the standard deviation of luminance values across non-white pixels. To describe how much an object stands out from the white background, we quantified its *intensity* as the mean of the pixel-wise luminance difference to the white background. Note that this property was previously referred to as “brightness,” but was renamed to intensity (i.e., inversed brightness). As intensity depends on both the luminance and number of non-white pixels (i.e., object size), we also provide a *normalized intensity* measure that is size-independent. To describe the spatial variations of luminance, we calculated the spatial frequency content with a bi-dimensional fast Fourier transform and a subsequent radial average of the two-dimensional power spectra. Thus, the *median power* quantifies variations in pixel luminance at different spatial scales, independent of their location in the image. In addition, *complexity* of an image was defined by the number or proportion (*normalized complexity*) of pixels representing contour outlines, as determined by a Canny edge detection algorithm (Canny, 1986) with adjusted parameters.

#### Macronutrients

Caloric information was estimated by students of nutritional science using the database https://fddb.info. Each food was given a kcal/100 g and total kcal value for the depicted portion. Ratings were pooled across two to five raters.

### Normative Ratings

#### Participants

Participants (*n* = 245) completed an anonymous online survey to provide normative data for the additional *food-pics* images (21.2% male, mean age = 31.4 years, 87.3% German; see Table 1 for detailed participant demographics). Participants were recruited through different university mailing lists; thus, the sample comprised students and employees alike. Participants who rated less than three food images were excluded from the analyses. The survey was available between December 2016 and February 2017. Participants were offered participation in a raffle for 5 × 30 Euros. The ethics board of the University of Salzburg approved this study.

**Table 1:** Subject demographics.

*N* = 245	*n* (%)	Mean (*SD*)	Median (Range)
Age (years)	–	31.4 (12.5)	27.0 (18–74)
**Gender**			
Male Female	52 (21.2%) 193 (78.8%)	–	–
**Nationality**			
Germany	214 (87.3%)	–	–
Austria	15 (6.10%)	–	–
Switzerland	2 (0.82%)	–	–
Other European country	7 (2.90%)	–	–
Non-European country	7 (2.90%)	–	–
Body Mass Index (kg/m^2^)	–	23.1 (4.35)	22.2 (16.4 – 44.0)
**Eating style**			
Omnivore	191 (78.0%)	–	–
Vegetarian	45 (18.4%)	–	–
Vegan	9 (3.67%)	–	–
**Current dieting behavior**			
Currently dieting Not dieting	27 (11.0%) 218 (89.0%)	–	–
**Employment**			
College/University^∗^	141 (57.6%)	–	–
Apprenticeship	43 (17.6%)	–	–
Self-employed	1 (0.41%)	–	–
Other	60 (24.5%)	–	–


*^∗^Studying psychology (85.0%), nutrition (2.92%), and other (11.4%).*

#### Online Survey

Participants provided demographic information on their age, gender, height, weight, occupation, and nationality as well as on eating habits (omnivore/vegetarian/vegan, dieting, or not dieting; see Table 1) before they rated the pictures. Each participant viewed and rated a random selection of 40 foods out of the 328 new food images, as participants could not have reliably rated all 328 images. Participants further rated five food images from the old *food-pics* database and a random selection of eight non-food images out of all 315 non-foods to check for comparability of the old and the new rating sample. Participants were given a detailed explanation of each of the scales and shown an example rating for all scales. *Familiarity* (German: “Bekanntheit”) was defined as whether the participant recognized the object or not. *Recognizability* (German: “Erkennbarkeit”) was defined as whether the object was easy or difficult to identify. *Complexity* (German: “Komplexität”) was characterized by “many components or details,” and “many colors/edges/pieces.” *Valence* (German: “Valenz”) was characterized by how negatively or positively the participant viewed the object; that is, whether they found it was repulsive or attractive. *Arousal* (German: “Erregung”) was characterized by how much the object aroused an emotional reaction in the participant. *Palatability* (German: “Schmackhaftigkeit”) was characterized by how delicious the participant found the depicted food in general, regardless of whether they wanted to eat it in the moment or not. *Desire to eat* (German: “Verlangen”) was characterized by how much the participant would like to eat the depicted food if it were available at that moment. Each image was displayed individually and participants were asked to rate each aspect of the depicted food. Response options for *familiarity* and *recognizability* were dichotomous (yes/no) and visual analog scales (VAS; solid horizontal bars approximately 8 cm long) with anchors on either extreme were used for ratings of *complexity* (“very little” to “very high”), *valence* (“very negative” to “very positive”), *arousal* (“not at all” to “extremely”), *palatability* (“not at all” to “extremely”), and *desire to eat* (“not at all” to “extremely”). Responses were provided via mouse click and ranged from 0 (leftmost extreme) to 100 (rightmost extreme); the value was not shown to participants.

## Results

### Normative Ratings

Each food image was rated by 14 to 47 participants (*M* = 28.21 images, *SD* = 5.26).

#### Interrater Reliability

Intraclass correlation coefficients (ICC) were then calculated using SPSS statistics (Version 24; IBM Corp.) based on a mean rating (*k* = 2), consistency, two-way random-effects model to compare normative ratings from the *food-pics* sample with normative ratings of the *food-pics_extended* sample. For food images, reliability was good for recognizability, familiarity, complexity, palatability, valence, and arousal (ICC = 0.870, 0.810, 0.801, 0.772, 0.834, and 0.820, respectively), and moderate for craving (ICC = 0.658). For non-food images, reliability was good for recognizability, familiarity, complexity, and arousal (ICC = 0.815, 0.850, 0.791, and 0.756, respectively), and moderate for valence (ICC = 0.671).

*Food-pics_extended* replaces *food-pics*, and images and metadata are available at http://food-pics.sbg.ac.at. Users of both *food-pics* and *food-pics_extended* are asked to cite the present report describing *food-pics_extended*.

### Overview Over Selected Food Image Databases

To guide researchers in selecting images according to their needs, we have compiled a table describing all of the above-mentioned datasets (see Table 2). Regarding set size, the following ordering emerged: *food-pics_extended*, *food-pics*, F4H, FRIDa, MaPS, OLAF, IAPS_foods. Normative ratings were available for all datasets, with the most ratings per image available for IAPS_foods, followed by F4H, *food-pics*, *food-pics_extended*, FRIDa/MaPS, and OLAF. Image characteristics were available for *food-pics*, *food-pics_extended* and FRIDa. Energy density is available for all datasets but OLAF and IAPS_foods.

**Table 2:** Overview of stimulus sets.

Database	Authors, Year	# Food images	# Non-food images	Sample # Participants Sample characterization • Normative ratings # Ratings per image	Image characteristics	Food characteristics	Image types	Comment strengths/weaknesses
*Food-pics*	Blechert et al. (2014)	568	315	German speaking adult sample: # Participants: 831 Age: 24.7 ± 5.5 (range: 18–65) 83.3 % female Predominantly from German-speaking countries (Austria, Germany, Switzerland) US-American adult sample: # Participants: 496 Age: 35.9 ± 13.4 (range: 18–77) 63.7% female Predominantly North American University of Hagen adult sample: # Participants: 638 Age: 32.8 ± 10.1 (range: 17–73) 82.8 % female Predominantly German Austrian underage sample: # Participants: 23 Age: 13.9 ± 1.6 (range: 11–18) 50.8 % female Predominantly Austrian • Palatability • Desire to eat • Valence • Arousal • Recognizability • Familiarity # Ratings per image: ∼49	• Colors • Brightness • Size • Contrast • Spatial frequencies • Complexity • Norm. complexity	• Energy density (calories), experimenter estimated • Macronutrients	Food images: • Fruits (76 images) • Vegetables (118) • Chocolate (65) • Fish (13) • Meat (63) • Nuts (10) • Drinks (9) Non-food images: • Flowers/leaves (42) • Animals (37) • Tools (23) • Non-kitchen household (89) • Kitchen utensils (46) • Office (20) • Food packaging (33)	• Wide range of foods • Focus on Western foods • Data from children and adults • Detailed macronutrient data • Open to add images
*Addition to food-pics* (Addition + *food-pics* = *food-pics_extended*)	This article	328 (896 total with f*ood-pics)*	0	# Participants (adults): 245 Age: 31.4 ± 12.5 (range: 18–74) 78.8 % female Predominantly from German-speaking countries (Austria, Germany, Switzerland) • Palatability • Desire to eat • Valence • Arousal • Recognizability • Familiarity # ratings per image: ∼28	• Colors • Intensity • Norm. Intensity (formerly brightness) • Size • Contrast • Spatial frequencies • Complexity • Norm. complexity	• Energy density (calories), experimenter estimated	All food images: • Fruit (64 images) • Vegetables (102) • Chocolate (30) • Fish (16) • Meat (49) • Nuts (5) • Drinks (2)	• Wide range of foods • Focus on Western, Asian and Middle Eastern food • Open to add images
FRIDa	Foroni et al. (2013)	295	582	# Participants (adults): 73 Age: 23.1 ± 3.3 (range: 18–30) 53.4 % female Predominantly Italian • Calories • Distance from edibility • Level of transformation • Valence • Arousal • Familiarity • Typicality • Ambiguity # Ratings per food-image: ∼5–14 # Ratings per non-food-image ∼8–21	• Size, • Brightness, • High spatial frequency	• Energy density (calories), rated	Food images: • Natural-food (99 images) • Transformed-food (153) • Rotten-food (43) Non-food images: • Natural-non-food items (53) • Artificial food-related objects (119) • Artificial objects (299) • Animals (54) • Scenes (57)	• Food from Mediterranean cuisine • Small sample size
F4H	Charbonnier et al. (2016)	370	41	Adult sample: # Participants: 449 Age: 33.7 ± 13.1 (range: n.a.) 70.2 % female Scottish, British, Dutch, Greek Underage sample: # Participants: 191 Age: 12.5 ± 2.3 (range: n.a.) 55 % female Dutch, German, Hungarian, Swedish • Recognizability • Liking • (Calories) • Healthiness # Ratings per image: Adult sample: ∼72–77 Underage sample: ∼44–59	none	• Energy density (calories), rated and experimenter estimated	Food images: • Snacks, fruits, vegetables and main meals on plates Non-food images: • Non-food objects on plates	• Data from children and adults • Pictures are taken in different regions of Europe • Country specific food and different preparations • Exclusively from Western countries • High degree of standardization. • Open to add images
IAPS_foods	Lang et al. (2008)	48	1148	# Participants (age: n.a.) • Valence • Arousal • Dominance # Ratings per image: ∼100	none	none	Food images: • Main meals • Desserts Non-food images: • IAPS images	• Large group of participants and raters per image • Small image number • Complex backgrounds • Image content several decades old
MaPS:	King et al. (2018)	144		# Participants (adults): 25 Age: 20.6 ± 1.1 (range: n.a.) 84 % female Predominantly North American • Interest • Appetite • Nutrition • Emotional Valence • Liking • Frequency # Ratings per image: 25	none	• Energy density (calories) • Macronutrients	• Foods with extreme values on fat, sugar, complex carbohydrate, and protein content	• Small sample size • Small image set size • fMRI data • Detailed macronutrient data
OLAF	Miccoli et al. (2014)	96	36 (IAPS)	# Participants (underage): 559 Age: 14.2 ± 1.4 (range: 11–17) 50.8 % female Predominantly Spanish • Valence • Arousal • Dominance • Craving # Ratings per image: 18	none	none	Food images: • Food compositions and complex arrangements • Fruits • Vegetables • Sweet high-fat foods • Salty high-fat foods Non-food images: • IAPS images	• Display of food images on comples backgrounds • Very close cutouts • “Eye-level” photos • Quality differs between the images (e.g., brightness) • Ratings comparable to IAPS


*N.a., information not available; FRIDa, foodcast research image database**;** F4H, full for heath image data base; IAPS, international affective picture system; MaPS, macronutrient picture system; OLAF, open library of affective foods.*

## Discussion

The present report presents the *food-pics_extended* image dataset, an addition to the *food-pics* stimulus set that added 328 images to the original 568 images (the extended set, thus, replaces *food-pics* and contains a total of 896 images). In the following, we describe *food-pics_extended* (aim *i*), characterize ours and each of the major food image sets with a focus on advantages and limitations (aim *ii*), and finally present a guideline for choosing between sets by ranking sets on various dimensions (aim *iii*).

Regarding aim *i*, *food-pics_extended* enlarges and complements the *food-pics* database (Blechert et al., 2014): besides the mere addition of images, we amended the normative data in a way that allowed compatibility with the normative data of *food-pics*. Our agreement/consistency data indicate that this process was successful: normative ratings by the new raters were largely comparable to those of *food-pics* as evidenced by good interrater agreement for a subset of images presented to both subject pools. This suggests that researchers can use images and normative data from both image sets and, thus, *food-pics_extended* subsumes and replaces *food-pics*, so users of “old” and “new” images should refer to *food-pics_extended*. Some caution should be given for craving ratings, for which agreement indices were lower, and which are known to be very state-dependent and fluctuate (Shiffman, 2000). Images in *food-pics* and *food-pics_extended* were selected under the following principles: (A) all foods were set on a white background, mostly without context (plates are shown where necessary), (B) high recognizability for most images (though for some foods in *food-pics_extended*, recognition might depend on cultural knowledge; Jensen et al., 2016), (C) high image quality and esthetic appeal. Single foods as well as full meals and different combinations of single foods are included. Normative ratings are available from several large samples (German-speaking and North American). Thus, researchers interested in investigating certain subpopulations (e.g., older US females), can extract the respective normative ratings from the database and use them to select images accordingly (e.g., on high vs. low palatability, given high recognizability). *Food-pics_extended* comes with 315 non-food control images that can be matched in terms of physical stimulus properties to the food images on ratings of valence and arousal as well as on image characteristics.

Regarding aim *ii*, in reviewing established image databases, it became clear that while IAPS (Lang et al., 2008) has been of undebated importance for standardizing stimuli across laboratories, it is very limited in the food context. Its focus lies on images that vary strongly in valence and arousal. Its advantages include the inclusion of a large database of valence and arousal ratings and its extensive use in the literature. Users aiming to include non-food IAPS images in their study should thus opt for these images or for OLAF for reasons of comparability of the normative ratings. Yet, these advantages are offset by several shortcomings: food images are few in number (48) and images are embedded in varying and complex backgrounds that might influence the neural response as a result of their overall image complexity. Furthermore, rating data do not include important information such as palatability ratings or data on calorie density.

An approach similar to that of the IAPS was taken by the authors of the OLAF (Miccoli et al., 2014). Explicitly referring to the IAPS database, the authors provide 96 images that parallel the complex and contextualized character of the IAPS: images are taken “on eye level,” full meals are shown with an overrepresentation of high-energy and highly palatable foods, images are meant to particularly appeal to the observers’ affective response, and normative data are given in relation to other categories of the IAPS (negative, neutral, and positive IAPS). As a result of the naturalistic, contextualized setup, it is difficult to control aspects of the food (macronutrient content, energy density), its components (only parts of the foods visible), and constituents (several main and side dishes, gravy, toppings, etc.). Normative data (valence, arousal, dominance, craving) from a large group of Spanish children and adults are available, resulting in 18 ratings per image.

FRIDa from Foroni et al. (2013) was the largest set at the time of publication with 582 food images representing mostly Western foods, with a slight bias toward Mediterranean foods. It was the first set for which quantitative measures of image characteristics (i.e., size, mean brightness, and high spatial frequency power) were available. Such image characteristics are known to influence behavioral response times and performance (Felipe et al., 1993; Mace et al., 2005; VanRullen, 2006; O’Donell et al., 2010) as well as neurophysiological responses (Pourtois et al., 2005; Schadow et al., 2007; Kovalenko et al., 2012). Therefore, providing information about image characteristics is important because they represent a potential confound for the comparison between groups of images, such as high caloric vs. low caloric food. They were also the first to include spoiled or rotten foods, allowing interesting comparisons within the food category but with varying valence/edibility (Becker et al., 2016). Their inclusion of natural and artificial non-foods further allow for interesting food/non-food contrasts. Clear advantages are set size, comparison categories, and rating data on “degree of food transformation,” “distance from edibility,” and calories, which are not available in any other image set. Disadvantages include the strict omission of plates (even for soups), which created edge artifacts for some images, limitation of their normative data to relatively few ratings (5–14 ratings) per image from respondents predominantly from Italy.

The database F4H by Charbonnier et al. (2016) includes 370 images by the time of this writing. It was the first image set to publish a standardized image protocol that would allow the community to extend the image set with comparable parameters. It focuses on individual foods (mostly between one to ∼30 pieces of one food on a plate) and on standardized presentation. This allowed the authors to provide exact estimates of calorie density along with the subjective ratings of participants. This standardized character, however, decreases the esthetic appeal and decontextualizes foods, which are often consumed in meals and compositions. F4H also includes 41 non-food images without any ratings. Food images represent foods from different Western countries. Strengths also include normative data from children and adults from seven European countries on healthiness, calories, and similarity of images with real food. The high level of control over food content allows for precise calculations of macronutrients for studies focusing on this aspect (however, no such data other than subjective calorie content are included). Limitations include the aforementioned de-contextualization, lack of image characteristics, a relatively small set of unrated non-food images, and the focus on European foods and European normative data.

Macronutrient picture system (King et al., 2018) is a rather small image set (144 images) with a specialized purpose: neurocognitive research on the neural representations of different macronutrients. Thus, foods are relatively homogenous (but extreme) with regard to fat, sugar, and protein content. Advantages include the presentation of functional magnetic resonance imaging (fMRI) data that show neural activation patterns for foods varying in macronutrient composition (sugar, fat, protein) and correspondence of image content with items in a food preference questionnaire (Geiselman et al., 1998) allowing the parallel investigation of habitual food consumption and neural correlates.

Regarding aim *iii* and a *guideline* for choosing between sets, our review illustrates that each of the presented databases has advantages but also limitations. Thus, each ranking of image sets has to been done in the light of the specific research question. One important attribute of any database is the *number and variety of available images*, because this affects many different research questions. For various reasons, such as variety of diets and culture, it seems important to not constrain image choice within a given set. Small sets run the risk of omitting typical and frequently consumed foods in a given geographical area (e.g., dark bread in Central Europe, rice dishes in Asia) or retraining variability within a given food category (e.g., salty snacks). Researchers interested in a large number of foods and/or different cultures may decide for one of the larger sets, such as *food-pics_extended*, F4H or FRIDa. A variety of items allows one not only to tap into a wide range of foods and potentially a wide range of cultures but also to match image subsets on other aspects. For instance, one may be interested in calorie density as an independent variable, but want to match stimulus groups on image characteristics (e.g., colors) and degree of processing, while keeping palatability comparable. This would require complex matching operations as these variables are sometimes correlated (Foroni et al., 2013; Blechert et al., 2014).

Almost equally important for a range of research questions is the amount of normative data provided. It requires extensive normative data to ensure reliable palatability matching from a population resembling the intended study sample. Accordingly, the size of image database is also related to another relevant choice dimension, namely, *cross-cultural validity/applicability* and *availability of normative data*. With regard to size and cross-cultural applicability, F4H and *food-pics_extended* would be the ideal sets, while researchers with a focus on Mediterranean diets and samples may also use FRIDa and OLAF. In the realm of neuroimaging, and particularly in electroencephalography (EEG) or magnetoencephalography (MEG) research and reaction time-based studies, researchers may consider recognizability and physical image characteristics such as complexity, brightness, and other attributes that might affect brain responses. For those, the choice might be between FRIDa and *food-pics_extended*. Regarding research focused on macronutrient content, MAPS and *food-pics_extended* would be recommendable. Drinks or food packaging are only available in *food-pics_extended* and *food-pics*. Researchers aiming to extend the data bases with images from their own labs may opt for open-ended stimulus sets such as F4H.

Certain limitations need to be kept in mind. First, our review was selective and might have overlooked some image sets. However, we aimed to review the most popular, free databases focusing on human appetite studies. Second, regarding *food-pics_extended*, even though we intended to include Middle Eastern and Asian foods, there is still ways to go to include typical foods from all major areas of the world. Also, drinks are underrepresented but may be important. Macronutrients are available for a subset of *food-pics_extended* (images of the former *food-pics*). In fact, pointing to the usefulness of such information for all images, recent research shows that both high fat and high carbohydrate content is more reinforcing than equicaloric foods with either high fat or high carbohydrate content (Difeliceantonio et al., 2018). Adaptations for different age groups may also be worthwhile. For example, a subset of *food-pics_extended* (images of the former *food-pics*) has been examined in US adolescents aged 12–17, where an average of 75% of foods were recognized. There are no data yet available for younger participants. This indicates that while certainly enough images are available with good recognizability, there is still room for improvement for some foods. Cultural differences were also documented for *food-pics* ratings in Portugal (Prada et al., 2017), pointing to the need for further validation. Due to elevated public awareness of the issue of nutritional health, normative ratings may have to be updated periodically. For example, more recent samples gave higher valence ratings for low calorie foods than the original *food-pics* sample, tentatively pointing in that direction (although confounded with cultural differences, see Prada et al., 2017). Future research might extend normative data, image breadth, and include 3D images for virtual reality and more high-resolution images in various formats. Importantly, the normative ratings for *food-pics_extended* were obtained from a relatively homogenous sample of predominantly female, German-speaking, and educated individuals in their 30 s. A representative database would require inclusion of other age groups (particularly younger aged youth and children), less educated groups, more males, and importantly, participants from other geographical regions.

## Data Availability

Publicly available datasets were analyzed in this study. This data can be found here: http://food-pics.sbg.ac.at.

## Ethics Statement

This study was carried out in accordance with the recommendations of the ethics board of the University of Salzburg with written informed consent from all subjects. All subjects gave written informed consent in accordance with the Declaration of Helsinki. The protocol was approved by the ethics board of the University of Salzburg.

## Author Contributions

JB wrote the introduction, parts of the methods, and discussion. AL and SP compiled the images and the data base. KO and SP conducted the questionnaire study and ran the statistical analysis. NAB optimized the image characteristic scripts. All authors edited and approved the last version of the manuscript.

## Conflict of Interest Statement

The authors declare that the research was conducted in the absence of any commercial or financial relationships that could be construed as a potential conflict of interest.

## Footnotes

1 Many thanks to our *food-pics_extended* contributors: Loïc P. Heurley, Université Paris Ouest Nanterre La Défense, Nanterre, France Jang-Han Lee, Chung-Ang University, Seoul, Korea; Gal Sheppes, Tel Aviv University, Tel Aviv, Israel Loukia Tzavella, Cardiff University, Wales, United Kingdom; Olga Pollatos, Universität Ulm, Ulm, Germany Vaibhav Tyagi, Plymouth University, Plymouth, United Kingdom

2 http://food-pics.sbg.ac.at
